# Effect of Focal Cartilage Lesions on Patient-Reported Outcomes After Anterior Cruciate Ligament Reconstruction: A 10-Year Nationwide Cohort Study of 7040 Patients

**DOI:** 10.1177/03635465251350398

**Published:** 2025-07-06

**Authors:** Stian Kjennvold, Svend Ulstein, Asbjørn Årøen, Magnus Forssblad, Lars Engebretsen, Jan Harald Røtterud

**Affiliations:** † Akershus University Hospital, Oslo, Norway; ‡ Institute for Clinical Medicine, University of Oslo, Oslo, Norway; § Oslo Sports Trauma Research Center, Oslo, Norway; ‖ Stockholm Sports Trauma Research Center, Karolinska Institute, Stockholm, Sweden; ¶ Oslo University Hospital, Oslo, Norway; # The Norwegian Knee Ligament Registry, Bergen, Norway; Investigation performed at the Department of Orthopedic Surgery, Akershus University Hospital, Lørenskog, Norway

**Keywords:** knee sports trauma, knee articular cartilage, anterior cruciate ligament

## Abstract

**Background:**

Focal cartilage lesions are commonly associated with anterior cruciate ligament injuries. The long-term effect of these lesions on patient-reported outcomes after anterior cruciate ligament reconstruction (ACLR) remains unclear.

**Purpose:**

To determine the effect of cartilage lesions—partial thickness (International Cartilage Regeneration and Joint Preservation Society grades 1 and 2) and full thickness (grades 3 and 4)—on patient-reported outcomes 10 years after ACLR.

**Study Design:**

Cohort study; Level of evidence, 3.

**Methods:**

The study included all patients with primary unilateral ACLR enrolled in the Norwegian Knee Ligament Registry and Swedish Knee Ligament Registry from 2005 through 2008 (n = 15,783). At a mean follow-up of 10.1 years (SD, 0.2), 7040 (45%) patients completed the Knee injury and Osteoarthritis Outcome Score (KOOS). Multiple linear regression analyses were used to evaluate any associations between concomitant cartilage lesions and KOOS outcomes 10 years after ACLR.

**Results:**

Of the 7040 patients available at 10-year follow-up, 1425 (20.3%) had partial-thickness cartilage lesions at the time of ACLR, and 495 (7.0%) had full-thickness cartilage lesions. Multiple linear regression analyses revealed that partial- and full-thickness cartilage lesions were associated with significantly inferior scores across all KOOS subscales as compared with patients without cartilage lesions. Patients with full-thickness lesions had less postoperative improvement after ACLR in all KOOS subscales as compared with patients with partial-thickness lesions.

**Conclusion:**

Patients with concomitant partial- or full-thickness cartilage lesions reported significantly worse outcomes in all KOOS subscales 10 years after ACLR as compared with patients without cartilage lesions.

Focal cartilage lesions frequently accompany anterior cruciate ligament (ACL) tears, with a prevalence of approximately one-quarter of the patients in extensive nationwide population-based registries.^15^ Focal cartilage lesions differ from generalized osteoarthritic changes, but it is well established that ACL ruptures and focal cartilage lesions can lead to early-onset osteoarthritis.^1,2,11,14,16^ Additionally, there is a growing body of evidence suggesting that the presence of concomitant focal cartilage lesions can substantially affect knee function and alter the trajectory of patient-reported outcomes after ACL reconstruction (ACLR).^4,8,9,12^ However, there is still limited evidence to date on the long-term effect of articular cartilage lesions on patient-reported outcomes after ACLR.

Previous studies from the Scandinavian Knee Ligament Registries have shown that focal cartilage lesions present at the time of ACLR can have a negative effect on short- and midterm patient-reported outcomes.^21,23^ The clinical relevance, however, was uncertain, and the adverse effects of concomitant cartilage lesion seemed to increase over time, leaving a knowledge gap about the long-term prognosis for these young patients. This underlines the importance of extended follow-up to better understand the effect of cartilage lesions over time and the clinical implications in ACLR.

Knowledge of the long-term prognosis for these combined injuries is important in optimizing choice of treatment and management of patient expectations. The main objective of this study is to assess the effect of concomitant cartilage lesions on long-term patient-reported outcomes after ACLR. In the present study, the hypothesis was that patients with ACL ruptures, along with partial- or full-thickness cartilage lesions, would report worse long-term Knee injury and Osteoarthritis Outcome Score (KOOS) outcomes when compared with patients without such lesions 10 years after ACLR.

## Methods

### Study Design and Data Collection

The current study is a binational prospective cohort study including patients from the Norwegian Knee Ligament Registry (NKLR) and Swedish Knee Ligament Registry (SKLR). The registries were established in 2004 (Norway) and 2005 (Sweden) and designed to collect information prospectively on all cases of knee ligament reconstruction surgery nationwide.^6^ The Swedish registry was based on the Norwegian registry to facilitate collaboration, and there are no major between-country differences with regard to demographics or treatment strategies. The registration in both countries is voluntary, but registry compliance has been reported at >90%.^6,13^

Before surgery and at 2, 5, and 10 years after ACLR, the patients complete the KOOS questionnaire. It contains 5 subscales related to pain, symptoms, activities of daily living, sport and recreation, as well as knee-related quality of life. Each subscale has several items to result in a score graded from 0 to 100, with 100 representing no knee problems. The KOOS is used to evaluate the effect of knee injuries or conditions on a patient’s well-being and function and is validated for cartilage lesions of the knee.^5^

Patient information such as age, gender, mechanism of injury, date of injury, and previous knee surgery is also collected. Information on perioperative findings, such as concomitant ligament or meniscal injury and any surgical procedures (including ACL graft choice), is then registered by the attending surgeon. Among the entities registered, cartilage lesions are described according to the International Cartilage Regeneration and Joint Preservation Society (ICRS) guidelines,^3^ where lesion size is dichotomized as ≥2 or <2 cm^2^. Lesion depth is graded from 1 to 4. Grades 1 and 2 indicate superficial cartilage damage, with surface irregularities that do not exceed 50% of cartilage thickness. Grades 3 and 4 represent deeper lesions, extending from >50% of cartilage thickness down to or into subchondral bone. Patients with >1 concomitant cartilage lesion were categorized according to the lesion with the highest ICRS grade.

### Study Population

The current study is a 10-year follow-up of all patients who underwent a unilateral primary ACLR and were included in the NKLR or SKLR between January 1, 2005, and December 31, 2008. During this time frame, 15,783 patients were prospectively included in the registries. The same patient cohort has been described in studies on the effect of concomitant focal cartilage lesions on patient-reported outcomes at 2 and 5 years after ACLR.^21,23^

**Figure 1.**
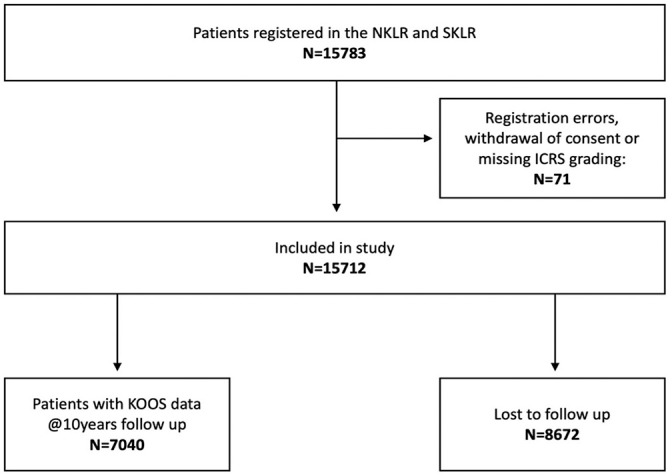
Study inclusion flowchart. ICRS, International Cartilage Regeneration and Joint Preservation Society; KOOS, Knee injury and Osteoarthritis Outcome Score; NKLR, Norwegian Knee Ligament Registry; SKLR, Swedish Knee Ligament Registry.

At the 10-year follow-up, 71 patients were removed for registration errors, missing data, or withdrawal of consent. Of the remaining 15,712 patients eligible for inclusion, 8672 had no 10-year patient-reported outcome measures and were considered lost to follow-up. This left 7040 (45%) patients in the study group (Figure 1).

### Baseline Characteristics

Baseline data are presented descriptively. Except for age and gender, the study group and those lost to follow-up demonstrated baseline equivalence in terms of preintervention status. The group lost to follow-up had a higher proportion of men and a younger age than the study group (Table 1).

**Table 1:** Baseline Characteristics at the Time of ACL Reconstruction for the Study Group vs Patients Lost to Follow-up*^*a*^*

	Median (Range), No. (%), or Mean ± SD	
	Study Group (n = 7040)	Lost to Follow-up (n = 8672)
Age at surgery, y	28 (8-69)	24 (10-66)
Time from injury to surgery, mo	8.9 (0-482)	9.0 (0-521)
Female sex	3403 (48.3)	3259 (37.6)
Previous ipsilateral knee surgery	1864 (26.5)	2263 (26.1)
Concomitant injury		
Ligament	528 (7.5)	583 (6.7)
Meniscal	3048 (43.3)	3764 (43.4)
Cartilage	1920 (27.3)	2232 (25.7)
ACL graft		
Hamstring tendon	5300 (75.3)	6895 (79.5)
Bone–patellar tendon–bone	1608 (22.8)	1598 (18.4)
Other/unknown	132 (1.9)	179 (2.1)
Preoperative KOOS		
Pain	75.0 ± 17.4	73.8 ± 18.0
Symptoms	71.6 ± 17.8	70.2 ± 18.2
Activities of daily living	83.8 ± 17.5	83.0 ± 17.4
Sport and recreation	42.2 ± 26.9	41.9 ± 27.2
Knee-related quality of life	34.1 ± 18.2	33.7 ± 18.2
Area of cartilage lesion		
<2 cm^2^	1113 (15.8)	1320 (15.2)
≥2 cm^2^	749 (10.6)	857 (9.9)
Not reported	58 (0.8)	55 (0.6)
ICRS grade of cartilage lesion		
1 or 2: partial thickness	1425 (20.2)	1715 (19.8)
3 or 4: full thickness	495 (7.0)	517 (6.0)

a ACL, anterior cruciate ligament; ICRS, International Cartilage Regeneration and Joint Preservation Society; KOOS, Knee injury and Osteoarthritis Outcome Score.

Patients included in the study were categorized as having no concomitant cartilage lesion, partial-thickness cartilage lesions (ICRS grades 1 and 2), or full-thickness cartilage lesions (ICRS grades 3 and 4). The baseline characteristics stratified by cartilage status are outlined in Table 2.

**Table 2:** Baseline Characteristics at the Time of ACL Reconstruction Categorized by Cartilage Status*^*a*^*

	Median (Range), No. (%), or Mean ± SD		
	No Cartilage Lesion (n = 5120)	Partial-Thickness Cartilage Lesions*^*b*^* (n = 1425)	Full-Thickness Cartilage Lesions*^*c*^* (n = 495)
Age at surgery, y	26 (8-69)	33 (13-67)	36 (14-64)
Time from injury to surgery, mo	8.0 (0-362)	12.2 (0-430)	15.8 (0-521)
Female sex	2565 (50.1)	629 (44.1)	209 (42.2)
Previous ipsilateral knee surgery	1119 (21.9)	532 (37.3)	213 (43.0)
Concomitant ligament injury	334 (6.5)	137 (9.6)	57 (11.5)
Concomitant meniscal lesion	2010 (39.3)	764 (53.6)	274 (55.4)
ACL graft			
Hamstring tendon	3845 (75.1)	1072 (75.2)	383 (77.4)
Bone–patellar tendon–bone	1169 (22.8)	337 (23.6)	102 (20.6)
Other/unknown	106 (2.1)	16 (1.1)	10 (2.0)
Area of cartilage lesion			
<2 cm^2^		888 (62.3)	225 (45.5)
≥2 cm^2^		487 (34.2)	262 (52.9)
Not reported		50 (3.5)	8 (1.6)
Location of cartilage injury			
Patella		584 (19.7)	788 (13.8)
Trochlea		172 (5.8)	35 (6,2)
Medial femoral condyle		994 (33.4)	288 (51.0)
Lateral femoral condyle		363 (12.2)	67 (11.8)
Medial tibial plateau		421 (14.2)	53 (9.4)
Lateral tibial plateau		438 (14.7)	44 (7.8)
Preoperative KOOS			
Pain	76.0 ± 16.7	73.4 ± 18.8	69.6 ± 19.3
Symptoms	72.5 ± 17.4	69.6 ± 18.1	67.8 ± 19.3
Activities of daily living	85.1 ± 16.6	81.3 ± 19.0	77.9 ± 19.7
Sport and recreation	43.9 ± 26.8	38.8 ± 26.9	35.4 ± 26.2
Knee-related quality of life	34.9 ± 18.1	32.7 ± 18.2	30.9 ± 18.3

a Blank cells indicate *not applicable*. ACL, anterior cruciate ligament; ICRS, International Cartilage Regeneration and Joint Preservation Society; KOOS, Knee injury and Osteoarthritis Outcome Score.

b ICRS grade 1 or 2.

c ICRS grade 3 or 4.

### Statistics

Stata SE Version 17.0 (Stata Corp LLC) was utilized for all statistical analyses. Differences were considered statistically significant for *P* values <.05. All crude mean KOOS outcomes, odds ratios, and regression coefficient estimates are presented with 95% confidence intervals.

To evaluate the potential effect of focal cartilage lesions on the 10-year patient-reported outcomes as measured by the KOOS, multivariable linear regression was employed. Separate analyses were made for each KOOS subscale, and the independent variables included in the regression model were sex, age at surgery (continuous variable), previous ipsilateral knee surgery (yes/no), concomitant ligament or meniscal injury (yes/no), and type of ACL graft (patellar tendon, hamstring, or other). To assess the effect of concomitant cartilage lesions, patients with no cartilage lesion at the time of ACLR were considered the reference population in all regression analyses. Partial- and full-thickness cartilage lesions were analyzed separately to compare the effect of lesion depth as graded by ICRS.

Notably, preoperative KOOS values and cartilage-specific characteristics, such as size and location, were deliberately not included as independent variables, as this would shift the focus of the regression model toward the effect of the ACLR rather than the cartilage injury. The results are presented with adjusted and unadjusted values to indicate the effect of each possible confounding factor.

To examine whether cartilage lesion size (<2 or ≥2 cm^2^) affected the 10-year KOOS results, separate analyses were conducted for patients with partial- and full-thickness cartilage lesions. A Wilcoxon rank sum test was used to evaluate any statistical significance.

To determine the proportion of patients with satisfactory or poor clinical outcomes, previously established KOOS threshold values for the Patient Acceptable Symptom State (PASS) and treatment failure (TF) were used.^10,19^ Patients had to exceed a specific threshold to reach PASS and fall below a certain threshold to be classified as TF. Patients with scores in between PASS and TF thresholds were categorized as “neither PASS nor TF.” The subscales analyzed were limited to KOOS knee-related quality of life and sports/recreation because they are regarded as the most relevant to describe the clinical state of patients with an ACL injury.^7,10^ For the KOOS quality of life and sport/recreation subscales, the predefined threshold values for the PASS were 73 and 72 points, respectively. For both subscales, the threshold for TF was 28 points.

## Results

At a mean follow-up of 10.1 years (SD, 0.2), 7040 (45%) patients completed the KOOS. The mean age at follow-up was 39.3 years (SD, 10.7). Of the 7040 patients, 1920 (27.3%) had 1 or more concomitant cartilage lesions at the time of ACLR. Among the 1920 patients with cartilage lesions, 1408 (73.3%) had no recorded treatment, while 223 (11.6%) underwent debridement, 105 (5.5%) underwent microfracture, and 26 (1.4%) received other procedures such as mosaicplasty or cell-based therapies. In total, 3334 cartilage lesions were identified, with 1425 (20.2%) patients having 1 or more partial-thickness lesions and 495 (7.0%) having 1 or more full-thickness lesions.

At the 10-year follow-up after ACLR, patients with partial-thickness cartilage lesions and patients with full-thickness cartilage lesions had inferior crude mean values on all KOOS subscales when compared with patients without cartilage lesions. Patients with full-thickness lesions had inferior crude KOOS outcomes for all subscales when compared with patients with partial-thickness lesions (Table 3).

**Table 3:** Crude Mean KOOS Outcomes Stratified by Cartilage Lesion Depth at 10-Year Follow-up After ACL Reconstruction*^*a*^*

	Mean (95% CI)		
	No Cartilage Lesion (n = 5120)	Partial-Thickness Cartilage Lesions*^*b*^* (n = 1425)	Full-Thickness Cartilage Lesions*^*c*^* (n = 495)
Pain	87.1 (86.7-87.5)	85.0 (84.1-86.0)	79.4 (77.5-81.2)
Symptoms	82.3 (81.8-82.8)	79.9 (78.9-80.9)	74.8 (73.0-76.7)
Activities of daily living	92.6 (92.2-92.9)	89.9 (89.0-90.7)	85.6 (83.9-87.3)
Sport and recreation	69.6 (68.9-70.4)	65.3 (63.8-66.8)	55.3 (52.4-58.1)
Knee-related quality of life	69.1 (68.4-69.7)	66.7 (65.4-68.0)	59.3 (56.9-61.8)

a ACL, anterior cruciate ligament; ICRS, International Cartilage Regeneration and Joint Preservation Society; KOOS, Knee injury and Osteoarthritis Outcome Score.

b ICRS grade 1 or 2.

c ICRS grade 3 or 4.

The multivariable linear regression analyses showed statistically significant inferior scores at the 10-year follow-up for all 5 KOOS subscales in patients with cartilage lesions as compared with the patients with no cartilage lesion (Table 4).

**Table 4:** Unadjusted and Adjusted Regression Analyses of the Associations Between KOOS Subscales and Cartilage Lesions by Depth at 10-Year Follow-up After ACL Reconstruction*^*a*^*

		Partial-Thickness Cartilage Lesions*^*b*^*			Full-Thickness Cartilage Lesions*^*c*^*		
KOOS Subscale	No.	β	95% CI	*P* Value	β	95% CI	*P* Value
Pain							
Unadjusted	7012	−2.1	−3.1 to −1.1	<.001	−7.7	− 9.3 to −6.2	<.001
Adjusted	6723	−1.4	−2.4 to −0.3	.010	−6.9	−8.5 to −5.2	<.001
Symptoms							
Unadjusted	7039	−2.4	−3.4 to −1.3	<.001	−7.5	−9.1 to −5.8	<.001
Adjusted	6747	−2.1	−3.2 to −1.0	<.001	−7.4	−9.1 to −5.6	<.001
Activities of daily living							
Unadjusted	7009	−2.7	−3.5 to −1.8	<.001	−7.0	−8.3 to −5.6	<.001
Adjusted	6720	−1.5	−2.4 to −0.6	.001	−5.3	−6.7 to −3.9	<.001
Sport and recreation							
Unadjusted	7030	−4.3	−6.0 to −2.7	<.001	−14.4	−17.0 to −11.8	<.001
Adjusted	6740	−2.6	−4.3 to −0.8	.004	−11.6	−14.4 to −8.6	<.001
Knee-related quality of life							
Unadjusted	7037	−2.4	−3.8 to −0.9	.001	−9.8	−12.0 to −7.5	<.001
Adjusted	6745	−2.0	−3.5 to −0.5	.010	−9.5	−11.9 to −7.1	<.001

a Adjusted for sex, age at surgery, previous ipsilateral knee surgery, concomitant ligament or meniscal injury, and type of ACL graft. Patients without cartilage lesion are the reference.

ACL, anterior cruciate ligament; β, regression coefficient; ICRS, International Cartilage Regeneration and Joint Preservation Society; KOOS, Knee injury and Osteoarthritis Outcome Score.

b ICRS grade 1 or 2.

c ICRS grade 3 or 4.

The crude KOOS results for all patients with concomitant cartilage lesions (ICRS grades 1-4) when stratified by lesion size ≥2 or <2 cm^2^ showed that the larger lesions predicted significantly inferior KOOS outcomes for all subscales at the 10-year follow-up (Table 5).

**Table 5:** Crude Mean KOOS Outcomes for Patients With Cartilage Lesions (ICRS Grades 1-4) by Area of Cartilage Lesion at 10-Year Follow-up After ACL Reconstruction*^*a*^*

	Cartilage Lesion Size, Mean (95% CI)		
KOOS Subscale	>2 cm^2^ (n = 1111)	<2 cm^2^ (n = 749)	*P* Value
Pain	84.9 (83.9-86.0)	81.5 (80.0-83.0)	<.001
Symptoms	79.8 (78.7-81.0)	76.6 (75.2-78.1)	<.001
Activities of daily living	90.1 (89.1.91.0)	86.8 (85.4-88.2)	<.001
Sport and recreation	65.7 (63.9-67.4)	58.3 (56.0-60.6)	<.001
Knee-related quality of life	66.1 (64.6-67.6)	60.9 (60.9-64.7)	.012

a ACL, anterior cruciate ligament; ICRS, International Cartilage Regeneration and Joint Preservation Society; KOOS, Knee injury and Osteoarthritis Outcome Score.

**Figure 2.**
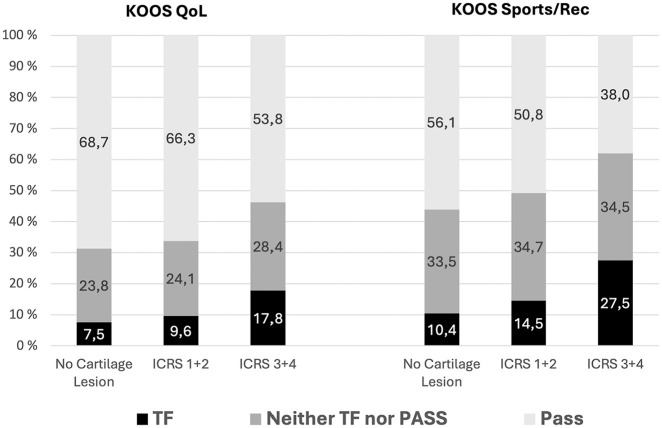
The proportion of patients reaching the PASS and treatment failure for the 2 KOOS subscales knee-related quality of life and sport/recreation when stratified by cartilage lesion depth. ICRS, International Cartilage Regeneration and Joint Preservation Society; KOOS, Knee injury and Osteoarthritis Outcome Score; PASS, Patient Acceptable Symptom State; QoL, quality of life; Sports/Rec, sport and recreation; TF, treatment failure.

PASS and TF threshold values have been established for patients undergoing ACLR.^10,19,25^ PASS and TF were used to establish the proportion of patients within the different clinical outcome categories. The highest proportion of failures and the lowest proportion of patients reaching the PASS were found in the full-thickness cartilage lesion group. The group differences were more pronounced for sport/recreation as compared with quality of life (Figure 2).

## Discussion

The main finding of the present study is that the presence of partial- and full-thickness cartilage lesions at the time of ACLR predicts inferior patient-reported outcomes as represented by KOOS outcomes 10 years after surgery. Patients with full-thickness cartilage lesions had significantly worse results than patients with partial-thickness lesions, and they had a higher proportion of patients defining their treatment as having failed.

Although smaller studies have reported no adverse long-term effects on patient-reported outcomes,^24,27^ we believe that our findings suggest a correlation between cartilage lesions and inferior outcome 10 years after ACLR. The results are consistent with the 2- and 5-year follow-up of the same cohort.^21,23^ The negative effect of concomitant cartilage lesion as compared with the “no cartilage lesion” group increased up to the 10-year follow-up. This is exemplified by the growing difference in mean KOOS quality of life in disfavor of the patients with full-thickness lesions when compared with those without cartilage lesions, increasing from 5.7 points at 2 years to 7.2 at 5 years and 9.8 at 10 years. One might assume that this is due to further cartilage degeneration over time, as studies have demonstrated increased risk of osteoarthritis in patients with full-thickness cartilage lesions.^2^ This is in line with a study by Visnes et al,^26^ who reported a higher incidence of knee replacement surgery at 15-year follow-up in patients with full-thickness cartilage lesions at the time of ACLR.

It is, however, worth noting that almost all crude mean KOOS results increased from the 2-year follow-up to the 10-year follow-up for all 3 groups. The exception is the sport/recreation subscale, which showed a decrease in mean scores from 2 to 10 years for the patients with partial- and full-thickness cartilage injury. This might be influenced partly by differences in age between groups, where patients with full-thickness cartilage injuries were 10 years older on average than patients without any cartilage injuries and therefore less sports active. Despite adjustment for this and other possible confounding variables in the regression analyses, a deterioration in KOOS sport/recreation outcome was still evident at the 10-year follow-up. Even at baseline, the patients with cartilage lesions had lower KOOS values in all subscales as compared with patients without such lesions, and this difference could account for most of the between-group difference at later follow-ups.

Although the minimal clinically important difference of the KOOS for this patient group is not firmly established, the between-group differences in some of the KOOS subscales are most likely clinically significant at the 10-year follow-up, at least for the full-thickness cartilage lesion group.^20^ The clinical significance is also supported by the magnitude of group differences in the proportion of patients exceeding the PASS and TF thresholds. Almost a third of the patients with full-thickness cartilage lesions reported a KOOS sport/recreation subscale outcome at or below the TF threshold value at the 10-year follow-up, indicating a poor outcome. In comparison, for the patients without cartilage lesions at the time of ACLR, about 10% had a KOOS sport/recreation outcome at or below the TF threshold. Conversely, 56% of patients without cartilage lesions reached the PASS for sport/recreation after 10 years, as compared with only 38% in the full-thickness cartilage lesion group. These are major group differences that should be part of patient information when cartilage lesions are encountered during ACLR.

This is, to our knowledge, the largest study to examine the long-term effects of cartilage lesions on patient outcomes after ACLR, providing robust statistical power. The 10-year follow-up allows for a comprehensive understanding of the effect of concomitant cartilage lesions on knee function over time. In addition, data in the registries are collected independently of the research question, and selection bias or recall bias is thus limited. The use of nationwide registries from 2 countries with a majority of the patient population included further strengthens the external validity of the findings.

The observational nature of the study design limits the ability to infer causality. Additionally, a rate of loss to follow-up exceeding 50% is a major limitation in the present study with a potential for attrition bias. Despite this, baseline analyses demonstrated minimal differences between groups, and adjustments for possible confounders, including age, gender, previous ipsilateral knee surgery, concomitant ligament or meniscal injury, and type of ACL graft, were made using multivariable regression models. Previous responder studies from the Danish Knee Ligament Registry and Hospital for Special Surgery also found that KOOS values were similar between responders and nonresponders, suggesting that the data may still be valid despite high attrition rates.^17,18^

The selection of independent variables was based on previous literature and clinical assumptions, but variables were limited to the entities listed in the registries. Other variables that could influence the results, such as body mass index, smoking status, and imaging data (eg, radiographs), were not available, which is a common limitation in registry-based research. This means that the current study could not track development of osteoarthritis, which could influence the patient-reported outcomes.

Our findings should be considered when informing patients with concomitant cartilage injuries at the time of ACLR. Currently, there is limited knowledge on the optimal way to treat these cartilage lesions, and our study cannot conclude that cartilage lesions should be treated at the same time as ligament reconstruction. However, Rotterud et al^22^ demonstrated superior short-term outcomes for debridement as compared with microfracture of focal cartilage lesions during ACLR. Efforts should be made to develop future recommendations for specific cartilage treatment according to the type of lesions.

## Conclusion

Patients with ACL injury with concomitant partial- and full-thickness cartilage lesions had inferior outcomes when compared with the patients without cartilage injuries, as reported by KOOS outcomes 10 years after ligament reconstruction. Patients with full-thickness lesions at the time of ACLR showed significantly worse scores for all KOOS subscales than patients with partial-thickness lesions.
